# SiO_2_-coated lead halide perovskites core–shell and their applications: a mini-review

**DOI:** 10.1098/rsos.230892

**Published:** 2024-01-31

**Authors:** Cuc Kim Trinh, Zubair Ahmad

**Affiliations:** ^1^ Chemical Engineering in Advanced Materials and Renewable Energy Research Group, School of Technology, Van Lang University, Ho Chi Minh City, Vietnam; ^2^ Faculty of Applied Technology, School of Technology, Van Lang University, Ho Chi Minh City, Vietnam; ^3^ School of Chemical Engineering, Yeungnam University, 280 Daehak-ro, Gyeongsan, Gyeongbuk 38541, Republic of Korea

**Keywords:** lead halide perovskites, core–shell, silica shell, environmental/chemical stability

## Abstract

Research on lead halide perovskites has demonstrated that they are one of the potential materials for optoelectronic and bio-related applications owing to their promising optical and electronic properties. However, their poor chemical stability in ambient environments is a critical factor that affects their practical applications. Silica is known for its excellent environmental/chemical stability and good optical properties. Therefore, SiO_2_-coated lead halide perovskites have been studied by introducing the protective layer containing SiO_2_ to prevent the rapid destruction of their surface chemistry and environmental degradation. It is found that lead halide perovskite core–shell can significantly improve the stability and preserve their high photoluminescence quantum yield. In addition, controlling the shell thickness is also important to produce effective and suitable inorganic halide perovskites core–shell for practical applications. This mini-review discusses the stability, synthesis method and applications of SiO_2_-coated lead halide perovskite core–shell. Furthermore, the effect of the SiO_2_ shell thickness on lead halide perovskite core–shell-based applications is also reviewed.

## 1. Introduction

In recent years, lead halide perovskite materials with the structure ABX_3_ (where A is caesium, B is a lead cation and X is a halide anion) exhibit numerous advantages such as high brightness, and high photoluminescence quantum yield (PLQY) with narrow size distribution, which can provide great potential in optoelectronic devices [1]. These lead halide perovskite-based various applications such as solar cells [2–9], photodetectors [10], light-emitting diodes (LEDs) [11–17] and low-threshold lasers [18–20], etc., have been increasingly investigated. Although they have excellent optical properties, the lead halide perovskite materials' sensitive surface chemistry with moisture, oxygen in the ambient environment, and organic polar solvents are still one of the major issues. These disadvantages hindered their applications [21–23]. Therefore, various effective methods need to be designed to modify the surface of lead halide perovskites with other stable materials to enhance the stability against the environment and chemicals for practical applications.

Many research groups have tried to study the encapsulation of lead halide perovskite materials with other materials as an inert protective layer to protect and stabilize halide perovskite core–shell. There are various types of shells such as metal oxides (SiO_2_, TiO_2_, AlO_x_, etc.) [24–27], organic polymers (polystyrene-block-poly-2-vinyl pyridine, etc.) [28], metal chalcogenides (ZnS, CdS, etc.) [29–31], perovskites (CsPbBr_x_, Cs_4_PbX_6_, CsPb_2_Br_5_, Rb_4_PbBr_6_, etc.) [32–35], inorganic (NaBr, etc.) [36] and organics (PABr, etc.) [37]. Many reports have used TiO_2_ [24], Al_2_O_3_ [27], polyhedral oligomeric silsesquioxane [38] and other materials to stabilize the structure of inorganic perovskites core–shell. However, most of the core–shell perovskites are large particles, whereas many halide lead perovskite nanocrystals (NCs) were covered with a layer of silica. This limitation hinders the application of these materials to practical applications. For example, in the case of bio-related fields, single nanoparticles are highly suitable to allow uptake by cells. In the application of LEDs, small particles are also highly recommended for preparing highly uniform films. In another work, the Li group also embedded quantum dots (QDs) in the organic polymer (polystyrene) [39], and QDs@polymer mostly retain their electrical and optical properties. However, these materials are only suitable for luminescent films or bulk materials. Therefore, the challenge of obtaining isolated perovskites in this case still needs to be addressed.

Among these types of shells, silica has been commonly used as a shell to encapsulate inorganic perovskites. It is a transparent inorganic material with good stability against environmental/chemical factors and good optical properties [40]. Silica-based tetramethyl orthosilicate (TMOS), tetraethyl orthosilicate (TEOS) and 3-aminopropyl-triethoxysilane (APTES) have been selected as one of the most effective protective layers for protecting inorganic perovskites. These materials are promising capping agents for inorganic perovskites and the precursors for silica matrix. These capping agents contain –silyl ether groups (–Si–O–R), which can undergo hydrolysis to form a –Si–O–Si– cross-linked network. This network will act as a protective layer to cover and protect perovskites from external factors such as oxygen, moisture and other harmful substances. Especially, in the case of APTES, the presence of the –NH_2_ group can effectively passivate the surface of QDs. This passivation process prevents the loss of their high PLQY. One important advantage of this type of silica is that it allows for easier control of the shell thickness. This advantage makes perovskite@SiO_2_ suitable for various optoelectrical and bio-related applications [41–44]. This control can be obtained by manipulating parameters such as hydrolysis time when water is used during reaction time, APTES/OAm ratios, or up-to-synthesis methods, unlike when using other types of protective materials.

The SiO_2_-coated perovskite core–shell can significantly enhance the environmental and/or chemical stability and perceive their original high PLQY [41–43]. However, there are still some challenges, such as the thickness of the formed shell or multiple perovskites incorporated into one shell. In the case of a thick SiO_2_ shell, this shell layer will hinder carrier injection due to the insulating property of the SiO_2_ shell [42]. It will affect the device performance based on these perovkites@SiO_2_. For example, in optoelectronic applications, the shell thickness should be thin enough for effective charge carrier transport. In addition, these materials-based applications in various fields have not been extensively investigated. In this mini-review, the works related to SiO_2_-coated lead halide perovskite core–shell with various types of shells containing SiO_2_, including the synthesis methods, their environmental and/or chemical stability, and applications are reviewed. In addition, this mini-review also discusses the appropriate shell thickness of lead halide perovskite@SiO_2_ core–shell for various practical applications (table 1).

**Table 1.** The shell thickness, type of SiO_2_ shell and applications of various lead halide perovskite@SiO_2_.

reference	shell thickness	type of SiO_2_ shell	application
[41]	thick	TMOS	no device (photonic materials)
[43]	thick	TMOS	bioimaging and drug delivery
[44]	thick	TMOS	fluorescent sensor
[45]	composite	TMOS	down conversion white LED device (photonic materials)
[46]	thick	TMOS	down conversion white LED device (photonic materials)
[47]	thick	TEOS	fluorescent label
[48]	—	APTES + TEOS	no device
[49]	thick	TEOS	laser
[50]	thick	TEOS	laser
[51]	thick	silica spheres + TEOS	no device
[52]	thick	silica spheres + TMOS	white LED device (photonic materials)
[53]	—	silica spheres + TEOS	white LED device (photonic materials)
[54]	thick	TEOS + HMDS	white LED device (photonic materials)
[55]	—	TMOS + BN	perovskite-converted LED
[56]	—	APTES	down conversion white LED device (photonic materials)
[57]	—	APTES	remote fim device (photonic materials)
[58]	∼ 5 nm	APTES	white mini-LED device (photonic materials)
[59]	—	APTES	no device (photonic materials)
[42]	ultra-thin (< 1.5 nm)	APTES	PeLED (opto-electronic materials)
[60]	ultra-thin (< 2 nm)	APTES	PeLED (opto-electronic materials)
[61]	∼ 1.5 nm	APTES	no device
[62]	(< 1.5 nm)	APTES	bio-imaging (photonic materials)
[63]	> 2 nm	APTES	down conversion white LED device (photonic materials)
[64]	—	APTES	no device (photonic materials)
[65]	thick	APTES	photoluminescent sensor

## 2. Si cross-linking reaction procedure

The –Si–O–Si– cross-linking network forms in the following steps. The Si cross-linking can be initiated by contacting H_2_O molecules that exist in air and/or organic polar solvents. After the –SiOR group is transformed to silanol in the presence of H_2_O molecules, –SiOH will react with –SiOR and/or –SiOH to form an Si–O–Si– cross-linking matrix [41,42].

## 3. SiO_2_-coated lead halide perovskites

### 3.1. Tetramethyl orthosilicate/tetraethyl orthosilicate-based lead halide perovskites core–shell

**Figure 1.**
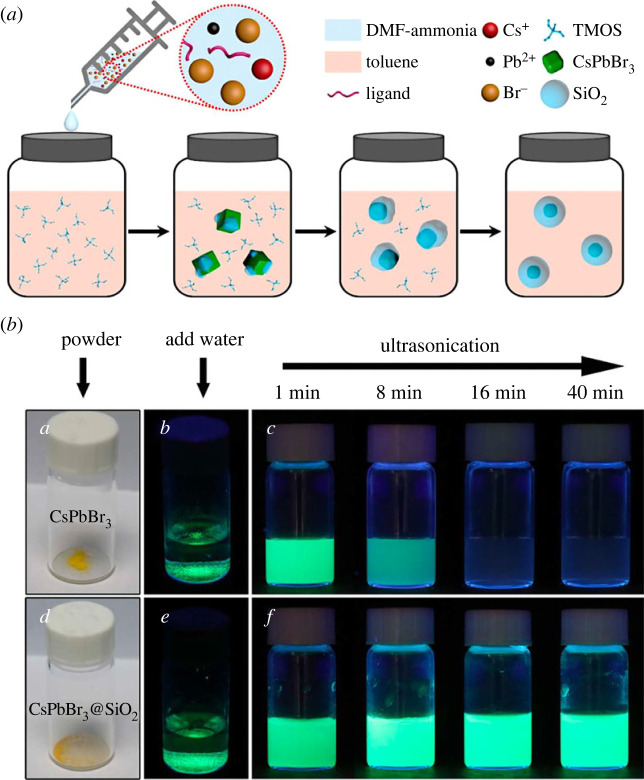
(*a*) The formation process of CsPbBr_3_@SiO_2_. (*b*) The stability of CsPbBr_3_ and CsPbBr_3_@SiO_2_ in water according to time. Adapted from [41] Copyright 2018 American Chemical Society.

Zhong *et al*. [41], Park *et al*. [43] and Chen *et al*. group [44] successfully reported the one-spot synthesis of CsPbBr_3_@SiO_2_ core–shell nanoparticles by adding caesium bromide, PbBr_2_, oleic amine (OAm), oleic acid (OA), dimethylformamide (DMF) and ammonia solution into toluene containing tetramethyl orthosilicate (TMOS) (figure 1*a*). This synthesis method was carried out without the requirement of an inert atmosphere and heat treatment. These characteristics can ensure better reproducibility and reduce costs in large-scale production. Thanks to the silica layer acting as a protective shell, the stability of CsPbBr_3_@SiO_2_ in humid air (25°C and humidity of 75%) and water was dramatically improved. The X-ray diffraction (XRD) signal of the SiO_2_-unprotected CsPbBr_3_ disappeared due to the decomposition of the core. In contrast to the XRD signal of CsPbBr_3_, the intensity of XRD peaks from CsPbBr_3_@SiO_2_ core–shell remains the same after 3 days. In the case of stability in water under harsh conditions (ultrasonication), the green emission of CsPbBr_3_ rapidly dropped. The bright emission completely disappeared after 40 min. However, the bright emission of CsPbBr_3_@SiO_2_ was still clearly observed after 40 min under ultrasonication. This result confirmed the improved stability compared with that of CsPbBr_3_ due to the successful protection of the SiO_2_ shell (figure 1*b*). In this study, a thick shell was obtained, which can affect carrier injection behaviour due to its insulating property. This study offers a simple and effective way to obtain highly stable CsPbBr_3_@SiO_2_ core–shell nanostructures in the inorganic-perovskites-related research area. While the Zhong group [41] focused on studying the effect of OAm and OA in controlling the shell thickness during the reaction, the Park group [43] applied this method to synthesize CsPbBr_3_@SiO_2_ core–shell with high luminescence and excellent water stability. These properties make CsPbBr_3_@SiO_2_ core–shell compatible for bioimaging and drug delivery applications, requiring significant water and environmental stability. A cytotoxicity test showed that these core–shell perovskite nanocrystals (PNCs) were bio-compatible with non-toxicity, making them suitable for cell imaging. Furthermore, CsPbBr_3_@SiO_2_ core–shell PNCs were also studied as fluorescent nanoprobes for bioimaging and drug-delivery applications. The result of this work suggests a suitable synthesis method for CsPbBr_3_@SiO_2_ core–shell PNCs with well-controlled shell thickness for biomedical-related applications. In addition, a fluorescent sensor array based on these CsPbBr_3_@SiO_2_ NCs was developed to detect sulfur-containing compounds (SCCs) such as benzothiophene (BT), dibenzothiophene (DBT), 2-methylbenzo[b]thiophene (2-MeBT), 3-methyl thiophene (3-MTP) and thiophene (TP) [44]. The formation of interactions between thiophene groups and -NH_2_ groups of CsPbBr_3_@SiO_2_ NCs resulted in a reduction in the fluorescence signal. This type of CsPbBr_3_@SiO_2_ NCs is a promising material for perovskite nanomaterial-based fluorescent sensors.

In contrast to the previous studies, water was used during the synthesis process to facilitate the hydrolysis step [45–47]. SiO_2_-coated CsPbX_3_ NCs were successfully reported [45]. A small amount of water was added to the hexane solution containing CsPbX_3_ NCs and kept at room temperature (RT) for 8 h. TMOS as the silica precursor was introduced into the hexane solution with CsPbX_3_ NCs. The presence of an amount of residual water in the hexane hydrolysed TMOS,

$$\begin{array}{rl} & \mathrm{Si}{\left({\mathrm{OCH}}_{3}\right)}_{4}+4H_{2}O\to \mathrm{Si}{\left(\mathrm{OH}\right)}_{4}+4CH_{3}\mathrm{OH}\\ & \mathrm{Si}-\mathrm{OH}+\mathrm{HO}-\mathrm{Si}\to \mathrm{Si}-O-\mathrm{Si}+H_{2}O. \end{array}$$

**Figure 2.**
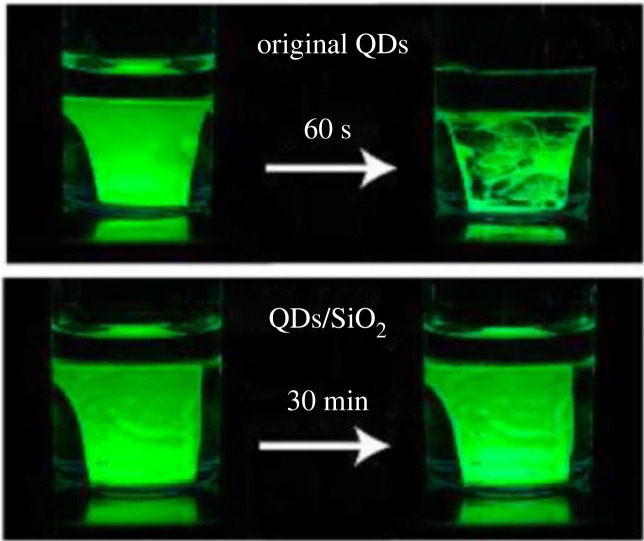
Photograph of images of CsPbBr_3_ and CsPbBr_3_@SiO_2_ immersed in water after 30 min. Adapted from [45] Copyright 2018 American Chemical Society.

The hydrolysis process of -OCH_3_ groups on TMOS led to the successful formation of an Si–O–Si shell on CsPbX_3_ NCs. It is found that the CsPbX_3_ NCs will decompose by the synergistic effect of oxygen and moisture if the hydrolysis time of TMOS is too long. The XRD pattern of CsPbBr_3_@SiO_2_ still maintained a cubic structure that was similar to that of CsPbBr_3_ NCs without a SiO_2_ shell, even after water treatment. In addition, the presence of CsPbBr_3_ NCs with SiO_2_ was confirmed by transmission electron microscopy (TEM) and high-resolution transmission electron microscopy (HRTEM) images along with energy-dispersive X-ray spectroscopy (EDS) mapping images. The interplanar distance of 0.58 Å was observed from HRTEM images. The UV–vis absorption and photoluminescence (PL) with a narrow peak (at 517 nm) of the CsPbBr_3_@SiO_2_ are observed. The photostability of CsPbBr_3_@SiO_2_ was much improved compared with that of CsPbBr_3_ NCs. After 24 h under ambient light, the PL intensity of CsPbBr_3_@SiO_2_ only dropped by 20%, whereas the PL intensity of CsPbBr_3_ without SiO_2_ protection decreased to 70%. An improvement in the stability against air and water of CsPbBr_3_@SiO_2_ was observed due to the protective effect of the SiO_2_ shell. The SiO_2_ shell effectively passivated surface defects, which contributed to enhanced stability. As shown in figure 2, the CsPbBr_3_@SiO_2_-based film still showed high PL emission after immersion in water for 30 min. By contrast, CsPbBr_3_-based film started to degrade after only 60 s in water. The stable CsPbBr_3_@SiO_2_ composite was used with K_2_SiF_6_: Mn^4+^ to fabricate a blue InGaN chip-based LED device with a wide colour gamut. The introduction of SiO_2_ shell-coated CsPbX_3_ (X = Cl, Br, I) perovskite NCs is an effective and simple method to produce CsPbX_3_ (X = Cl, Br, I) perovskite NCs-based optoelectronic device with high stability and good performance.

**Figure 3.**
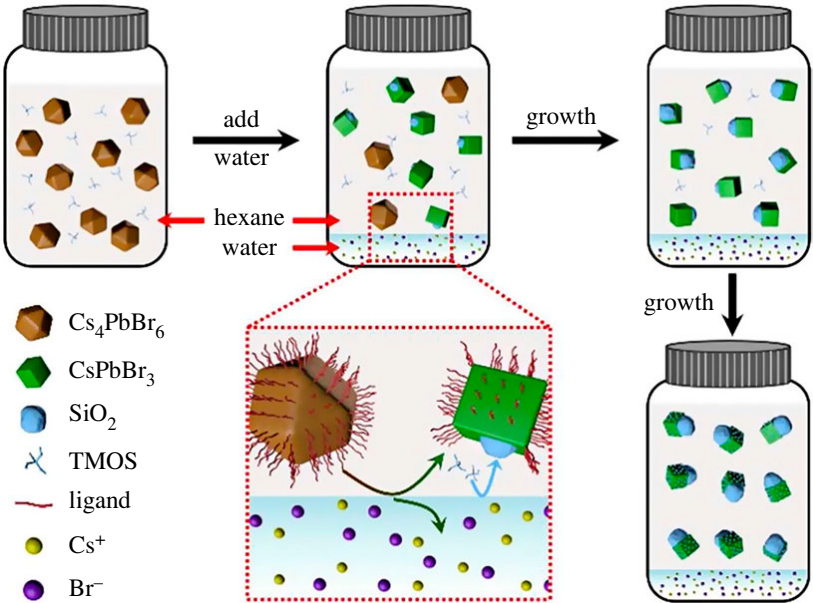
Schematic of the process of formation CsPbBr_3_@SiO_2_ Janus NCs. Adapted from [46] Copyright 2018 American Chemical Society.

Similar to the case of the Xia *et al*. group's research [45], the sol–gel method was also applied by Yin *et al*. group [46] to synthesize CsPbBr_3_@SiO_2_ NCs. After tetramethoxysilane (TMOS) was added into Cs_4_PbBr_6_ NCs with hexane solution, the amount of DI water was quickly injected into this solution. This mixture was kept under ambient conditions for 12 h. Finally, the precipitates were removed to collect the product after the centrifugation step (figure 3). When the reaction time was increased, more Cs_4_PbBr_6_ NCs were changed into CsPbBr_3_ NCs. Besides, the size of SiO_2_ also was proportionally increased with reaction time. The size of SiO_2_ is from approximately 3.4 nm to approximately 12.6 nm according to the amount of TMOS that was added during the reaction. However, if the amount of TMOS exceeds the limit, it will lead to the formation of free silica molecules. These free silica molecules can affect their optical and electronic properties due to their insulating property. Thanks to the protection of the silica layer, the stability of CsPbBr_3_@SiO_2_ NCs in the air, water and light treatment was dramatically enhanced compared with that of CsPbBr_3_. The CsPbBr_3_@SiO_2_ NCs were still stable in the water treatment for 7 days. In addition, their emission still showed green. The particle size of CsPbBr_3_@SiO_2_ NCs is much smaller than that of other reports, resulting in better dispersity in the solvent. The CsPbBr_3_@SiO_2_ NCs thin films were successfully fabricated due to their excellent dispersion in the solvent, small-size NCs and high stability. The WLED devices fabricated from CsPbBr_3_@SiO_2_ NCs combined with CdSe NCs and blue-emissive GaN LED chips obtained a 138% colour gamut of the National Television System Committee (NTSC, 1913) standard. This work shows that CsPbX_3_@SiO_2_-based nanomaterial is a promising material in practical applications such as white light devices application. It also provides a novel and simple approach to modifying the surface of perovskite nanocrystals with single particles and high stability, as well as controlling the shell thickness. Other SiO_2_-coated lead halide perovskite materials were also successfully produced by Maquieira *et al*. group [47]. This group successfully studied a novel and effective synthesis for monodispersed SiO_2_-coated CsPb_2_Br_5_ perovskite nanoparticles by controlling the chemical transformation of pre-synthesized CsPbBr_3_ in the presence of TEOS, ammonia and water. The blue emission of the core–shell perovskite nanoparticles could still be clearly observed after dispersion in water for 3 days. By optimizing ammonia/water ratios and reaction time, spherical CsPb_2_Br_5_@SiO_2_ core–shell NPs exhibited monodispersed morphology with suitable particle size and excellent water resistance for biosensing and bioimaging-related applications. Owing to these excellent properties, the core–shell nanoparticles were used as a fluorescent label to define bovine serum albumin (BSA). BSA was employed as a representative target protein.

**Figure 4.**
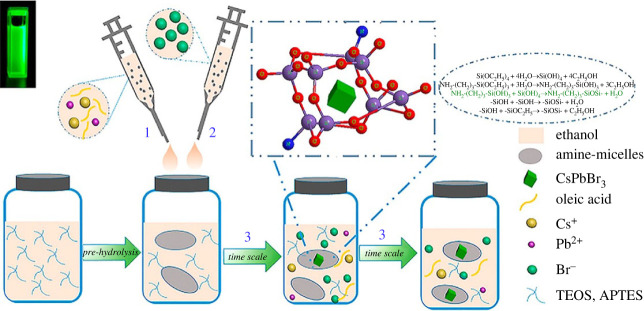
The illustration of the room-temperature synthesis process of CsPbBr_3_@SiO_2_ within APTES and TEOS in ethanol. Adapted from [48] Copyright 2020 American Chemical Society.

In addition, the combination of APTES and TEOS was found to be effective in the construction of silica micelles, which offered well-protected CsPbBr_3_ perovskite materials. CsPbBr_3_@SiO_2_ perovskites were synthesized by a novel room-temperature synthesis method [48] (figure 4). The controlling –NH_2_ group and –Si–O–Si–/–Si–OH in silica micelles is the main factor during the chemical reaction process. The final CsPbBr_3_@SiO_2_ exhibited a photoluminescence quantum yield of 61.9% and long-term stability in ethanol. The PL stability of the CsPbBr_3_@SiO_2_ ethanol sol was investigated. The bright green PL of CsPbBr_3_@SiO_2_ ethanol sol still persevered for 34 days and without the occurrence of a red-shift phenomenon in the PL spectra. These results indicated the effectiveness of amine-micelles in high PL stability of CsPbBr_3_@SiO_2_ sol. In summary, this work opens an effective strategy for the synthesis of long-term-stable inorganic halide perovskites in a highly polar solvent, which will be useful for various practical applications.

### 3.2. Silica spheres with tetramethyl orthosilicate/tetraethyl orthosilicate-based lead halide perovskites core–shell

Instead of using conventional TEOS or TMOS as a protective layer, Leng *et al*. group [49,50] encapsulated CsPbBr_3_ QDs into waterless silica spheres based on TEOS and applied CsPbBr_3_@SiO_2_ perovskites for laser applications. The hot injection method was used for synthesizing CsPbBr_3_ colloidal QDs, followed by injection of the silica precursor TEOS for the growth of silica layers. After several minutes, SiO_2_ layers successfully covered the surface of the CsPbBr_3_ QDs. In this work, CsPbBr_3_ QDs and CsPbBr_3_@SiO_2_ NCs exhibited similar PL spectra with a narrow green emission peak (at 522 nm) and narrow full width at half maximum (FWHM) of 18 nm. This indicated that the existence of the silica layer did not affect the optical properties. Enhanced stability in water of coated perovskite QDs by SiO_2_ was observed. CsPbBr_3_@SiO_2_ QDs in water still showed green light emission after 12 h, whereas for CsPbBr_3_ QDs green light emission completely disappeared. In addition, the PL decay rate of CsPbBr_3_@SiO_2_ film was slower than that of CsPbBr_3_ film, which demonstrated enhanced moisture resistance due to the existence of the SiO_2_ shell. To demonstrate the stability of CsPbBr_3_@SiO_2_ QDs for lasing devices, the operation of amplified spontaneous emission (ASE) of CsPbBr_3_ and CsPbBr_3_@SiO_2_ under continuous excitation with pump intensity of 600 µJ cm^−2^ in the air over 12 h was compared. After 12 h of continuous excitation, the result showed that CsPbBr_3_@SiO_2_ NCs maintained approximately 95% of light emission intensity, whereas the light emission intensity of CsPbBr_3_ QDs drastically decreased to 85%. This performance indicated that the silica spheres-coated perovskite QDs performed longer operating lifetimes under the atmosphere. Furthermore, CsPbBr_3_ QDs' optical properties exhibited stable ASE. Recently, the composites based on CsPbBr_3_ or CsPbCl_3_ perovskites and silica microspheres (MSs) synthesized using TEOS or TMOS as precursors are introduced to produce highly luminescent and stable mSiO_2_-wrapped perovskites (CsPbX_3_/MSs) for optoelectronic applications [51–53]. Owing to protection from well-ordered silica microspheres, these CsPbX_3_/MSs perovskites displayed water-resistant and thermal ultra-stability. Besides, these perovskites' optical properties still remain in the environment for a few weeks. These mSiO_2_-wrapped perovskites showed potential as materials in the white light-emitting diode (WLED) device. The performance of WLED devices achieved significant enhancement due to the good stability and optical properties of mSiO_2_-wrapped perovskites. Introducing a silica layer to the perovskite QDs is an effective strategy to weaken the degradation potential of perovskite nanomaterials. These studies provide a new direction for the development of perovskites/SiO_2_ with excellent stability against the environment, which are attractive for stable and high-performance photovoltaic and light-emitting device applications.

### 3.3. Tetramethyl orthosilicate/tetraethyl orthosilicate-based lead halide perovskites core–shell with surface modification

**Figure 5.**
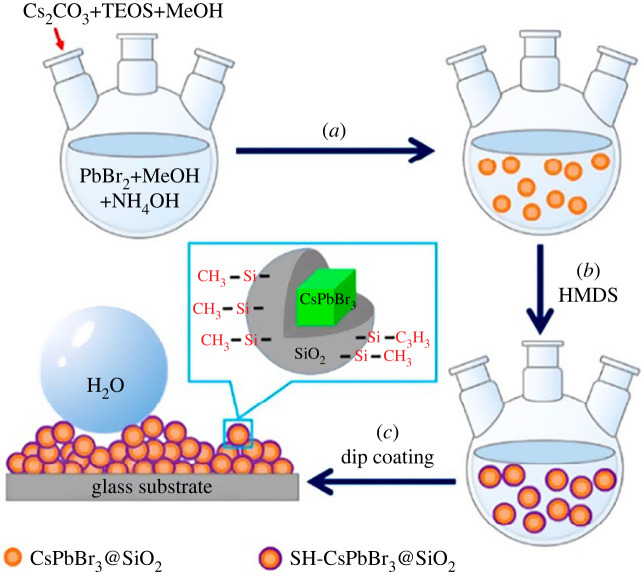
The illustration of the synthesis process of superhydrophobic CsPbBr_3_@SiO_2_ with TEOS. Adapted from [54] Copyright 2021 American Chemical Society.

As discussed in previous work, SiO_2_-coated CsPbBr_3_ perovskites effectively protect materials against external factors. However, TMOS and TEOS-based silica shells do not contain any functional groups that can be easily modified to be compatible with other materials or device fabrication. Therefore, surface modification of CsPbBr_3_@SiO_2_ perovskites is required to enhance their compatibility with various applications [54,55]. Xie *et al*. group [54] modified the surface of CsPbBr_3_@SiO_2_ perovskites as required to enhance their compatibility with various applications [54,55]. Xie *et al*. group [54] modified them by hydrophobic groups such as hexamethyldisilazane (HMDS) to produce superhydrophobic CsPbBr_3_@SiO_2_ nanoparticles and film (figure 5). The superhydrophobic SiO_2_-coated CsPbBr_3_ (SH-CsPbBr_3_@SiO_2_) was synthesized by combination hydrolysis of TEOS in the presence of NH_4_OH with surface modification by HMDS. Owing to the superhydrophobicity of the hydrophobic surface, SH-CsPbBr_3_@SiO_2_ films exhibited exceptional stability against water, heat and self-cleaning property. The hydrophobic surface can prevent various contaminants (dust, dirt, etc.) and help SH-CsPbBr_3_@SiO_2_ films clean and retain their optical properties. These properties make this type of perovskite highly suitable for optoelectronic applications, especially those used in outdoor environments. Meanwhile, thermally conductive surface-encapsulated CsPbBr_3_ PNCs were reported by Xu *et al.* group [55]. These CsPbBr_3_ PNCs prevented thermal-induced degradation caused by the low thermal conductivity of the SiO_2_ layer. Importantly, the thermal degradation will hinder their practical application. Therefore, PNCs-SiO_2_-boron nitride (PNCs-SiO_2_-BN) was newly synthesized by incorporating CsPbBr_3_ perovskite nanocrystals into assembled BN nanoplatelets through SiO_2_ cross-linking using TMOS. Owing to the high thermal conductivity, BN nanoplatelets minimize the heat accumulation on perovskite nanocrystals in light-emitting diodes. PNCs-SiO_2_-BN exhibited good thermal stability due to the assembly structure of PNCs-SiO_2_-BN which resulted in the effective protection of PNCs at high temperatures. PNCs-SiO_2_-BN-based PcLED displayed excellent stability at approximately 0.15 W cm^−2^ during 1000 h of sustained illumination.

### 3.4. 3-aminopropyl-triethoxysilane-based lead halide perovskites core–shell

The silica-based coating on APTES has been increasingly chosen as an effective protective layer for lead halide perovskites NCs. The use of APTES as a capping agent offers many advantages in the synthesis and stabilization of perovskite NCs. APTES facilitates the dissolution of PbX_2_ and enhances the stability of the inorganic perovskites.

Yu *et al*. [56], Lin *et al*. [57] and Xie *et al*. group [58] applied a modified hot injection method to the synthesis of perovskite QDs. After adding PbBr_2_, oleic amine, oleic acid and APTES into the 1-octadecene (ODE) solution, the caesium oleate solution was injected into this solution. Then, the flask was exposed to the air at a temperature of 20°C and stirred for 3 h. During this step, APTES will undergo slow hydrolysis,

$$\begin{array}{rl} & \begin{array}{c} -{\mathrm{SiOC}}_{2}H_{5}-+H_{2}O\;\to -\mathrm{SiOH}+C_{2}H_{5}\mathrm{OH}\;\;\;\\ -\mathrm{SiOH}+-{\mathrm{SiOC}}_{2}H_{5}\to -\mathrm{Si}-O-\mathrm{Si}-+C_{2}H_{5}\mathrm{OH}\; \end{array}\\ & \;-\mathrm{SiOH}+-\mathrm{SiOH}\to -\mathrm{Si}-O-\mathrm{Si}-+H_{2}O. \end{array}$$

The –NH_2_ group from APTES efficiently passivates the surface of perovskite NCs, preserving their high PLQY. The hydrolysis of the silyl ether groups results in the formation of a cross-linked matrix, encapsulating the perovskite NCs to achieve highly stable core–shell perovskite NCs against external factors. The APTES was used as a capping agent for perovskite QDs and a precursor for a silica matrix. The reaction process for the formation of perovskite QDs was carried out without the presence of even small amounts of water, which will prevent the decomposition of perovskite QDs. The successful formation of a shell was confirmed by Fourier-transform infrared spectroscopy (FT-IR). The existence of peak vibration of –Si–O–Si– and a weak peak of Si-OH is related to the hydrolysis of APTES, confirming the successful formation of a SiO_2_ cross-linked network. Owing to the SiO_2_ shell layer, green QD/silica almost maintained the same value of origin PLQY after a long period in the atmosphere at room temperature. In Yu [56] and Xie's work [58], the QD/silica was used as a photonic material, which was combined with a blue LED chip to fabricate a WLED. The CsPbBr_0.96_I_2.04_@SiO_2_ nanocrystals exhibited excellent optical properties and good stability against moisture, heat and light due to the protection of SiO_2_ shell. This material, with its excellent properties such as high stability, high PLQY and anion-exchange reactions, demonstrates great potential for white lighting applications. Inspired by the work of Yu *et al*. [56], the modified hot injection method was also used to produce silica-coated perovskites nanocrystals (SP-PNCs) with APTES, grafted onto the surfaces of PNCs [59]. The SP-PNCs exhibited high PLQY (80%) and were well-dispersed in various non-polar solvents. The well-dispersed SP-PNCs could be precipitated from their original solvents. In addition, they still retained their crystal structure, surface properties and photoluminescence quantum yield. This work provides a new greener method to synthesize SiO_2_-coated perovskite nanocrystals by precipitating step with polar solvents. However, in these studies, multiple particles with various sizes were encapsulated into the same SiO_2_ shell. This hindered these materials in some applications, such as bio-related fields, and LED films which require a single particle.

**Figure 6.**
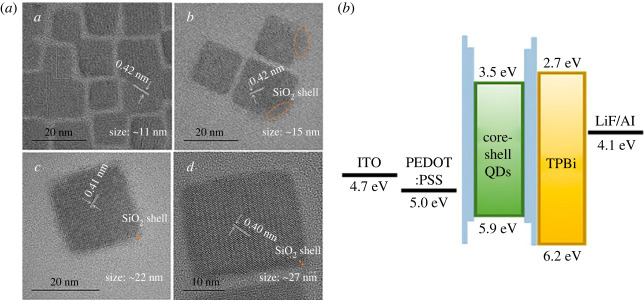
(*a*) TEM images of pristine CsPbBr_3_ and CsPbBr_3_@SiO_2_ QDs with different ratios of APTES/OAm. (*b*) Energy levels of materials and devices. Adapted from [42] Copyright 2021 American Chemical Society.

To overcome the issue of multiple particles and achieve an ultrathin SiO_2_ shell, Lee *et al*. group [42] synthesized green CsPbBr_3_@SiO_2_ core–shell QDs with APTES as SiO_2_ shell precursor by modified hot injection method with a one-step reaction. Firstly, PbBr_2_, oleic amine, oleic acid, and APTES were added into the ODE solution in an inert atmosphere. Secondly, the caesium oleate solution was injected into this solution at a temperature over 100°C. Then, the flask was exposed to the air for the hydrolysis step of the silyl ether groups in the APTES molecules to form SiO_2_ shell on QDs. This hot injection method is popularly applied to produce QDs, because it is one of the most effective synthesis methods to produce uniform QDs with narrow size distribution. Importantly, this group successfully investigated various APTES/OAm ratios during the reaction to achieve an ultrathin shell, where each particle was covered by a single shell. This allowed it to be used as an emissive layer in LEDs application. TEM data (figure 6*a*) showed that the obtained CsPbBr_3_@SiO_2_QDs exhibited uniform size and a controlled insulating shell thickness (less than 1.5 nm). This shell thickness was sufficiently thin to exhibit effective carrier injection behaviour. The surface of QDs was effectively passivated by the –NH_2_ group on APTES, which maintained high photoluminescence quantum yield with PLQY approximately 70%. As the APTES/OAm ratio increased, the PLQY of CsPbBr_3_@SiO_2_ QDs gradually increased, whereas the broadness of the PL peak decreased. The SiO_2_ shell effectively prevented polar solvents' penetration into perovskite quantum dots, resulting in excellent chemical and environmental stability. After 10 min of adding IPA/ethanol/water into perovskites QDs, the PL intensity of CsPbBr_3_ QDs without SiO_2_ shell rapidly reduced due to fast desorption of the oleylammonium bromide. By contrast, the water resistance and ethanol resistance of CsPbBr_3_@SiO_2_ core–shell QDs were much stronger those that of pure CsPbBr_3_. This work represents the first report of successful all-solution-processed perovskite QD-light-emitting diode (PeLED) fabrication using perovskite–metal oxide core–shell QDs as an emissive layer. Perovskite–metal oxide core–shell QDs had not previously been applied to PeLEDs due to the high carrier injection barrier of the metal oxide shell, even though it guarantees high chemical and environmental stability. However, this work successfully achieved PeLEDs using perovskite–metal oxide core–shell QDs (figure 6*b*) with a luminance of approximately 3200 cd m^−2^ owing to the ultrathin shell. The CsPbBr_3_@SiO_2_-based hole-only devices (HODs) demonstrated good charge injection efficiency with a hole mobility of approximately 1.39 × 10^−3^ cm^2^ V^−1^ s^−1^. This approach is not limited to green perovskite QDs and can be extended to any type of perovskite QDs. Furthermore, the core–shell perovskite QDs also show high potential for use in various solution-processed optoelectronic applications. The introduction of these perovskite/SiO_2_ core–shell QDs is a simple and effective method to produce new perovskite core–shell with the ultrathin shell, which is useful for perovskite QD-based optoelectronic devices.

**Figure 7.**
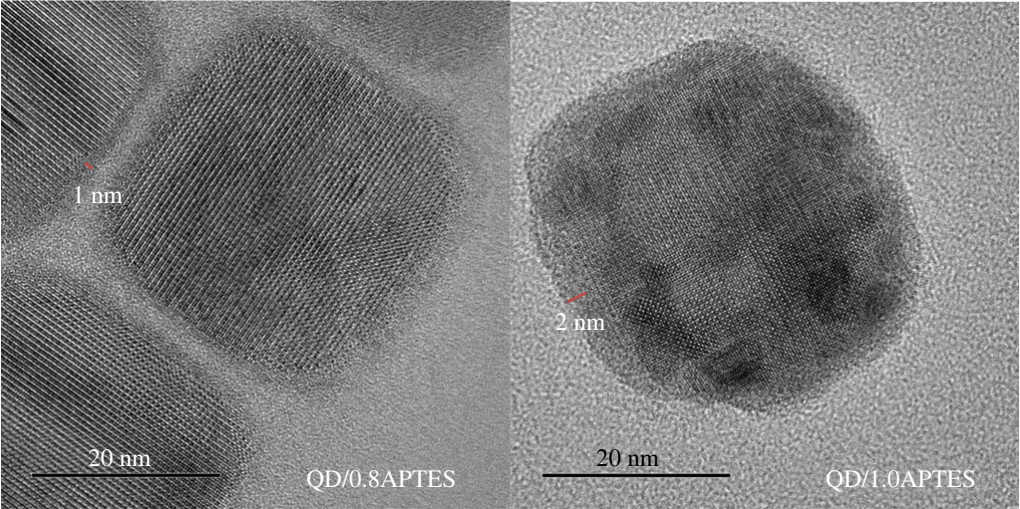
High-resolution TEM images of QD/0.8APTES and QD/1.0APTES. Adapted from [60] Copyright 2021 Royal Society of Chemistry.

**Figure 8.**
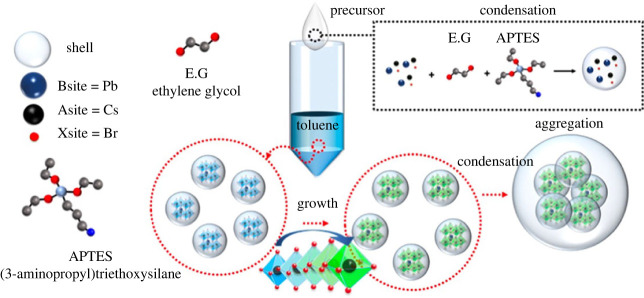
The formation process of the CsPbBr_3_@SiO_2_ QDs. Adapted from [61] Copyright 2022 American Chemical Society.

In addition, Lee *et al*. [60] also reported red CsPbI_3_@SiO_2_ core–shell QDs with APTES as the shell via a modified hot injection method. Owing to successful control over the shell thickness and gradient I doping, CsPbI_3_@SiO_2_ core–shell QDs could be applied to optoelectronic devices. Based on the result of TEM data, the SiO_2_ shell thicknesses of QD/0.8APTES and QD/1.0APTES were approximately 1 nm and approximately 2 nm, respectively, which are thin enough to exhibit effective carrier injection behaviour (figure 7). The mechanism of degradation of red perovskite under electric fields was investigated to solve the problem of the low operational stabilities of PeLEDs. The operational stability of the red perovskite core–shell QDs-based PeLEDs (QD/0.8APTES) was approximately 5000 times compared with those of red pristine-based PeLEDs. This indicated their high stability against temperatures and electric fields due to the protection of the SiO_2_ shell, which resulted in effectively passivating the surface defects. These studies provide a useful strategy for designing new red perovskite/SiO_2_ core–shell QDs with the ultrathin shell for optoelectronic devices. They demonstrated the potential to have a breakthrough in perovskite QD-based electronics such as LEDs, solar cells, thin film transistors and other optoelectronic applications. This study is not only applied to red perovskites, it can also be extended to other types of perovskites with different anions. In contrast to the synthesis method used by Lee *et al*. [42,60], Yoon *et al*. [61] employed a modified ligand-assisted reprecipitation technique to encapsulate CsPbBr_3_ perovskites QDs with an ultrathin silica shell (APTES) that would facilitate effective charge transport (figure 8). The *N*,*N*-dimethylformamide solution containing the perovskite precursors was poured into an antisolvent (toluene) to induce the precipitation of perovskite quantum dots. Afterwards, their precipitate was put in ODE at 225°C. This resulted in the formation of a silica protective layer against water. Owing to the narrow shell thickness of approximately 1.5 nm, this SiO_2_ shell could be useful for the electron/energy process from the core to the electron transport media through the Dexter energy transfer or electron transfer. Therefore, the transfer process should be used for the application of CsPbBr_3_@SiO_x_ perovskite QDs in various areas such as optoelectronic devices and solar-driven chemistry. This study provides valuable insights into the core–shell perovskites covered with silica shells in renewable energy applications.

To enable the utilization of perovskite materials in bio-related applications, Song *et al*. [62] synthesized CsPbBr_3_@SiO_2_ nanoparticles by using water during the reaction, instead of using the hot injection method without the presence of water. The SiO_2_ shell thickness was easily controlled by the hydrolysis process time. The synthesis process of CsPbBr_3_@SiO_2_ nanoparticles has two steps. The water was added into a solution containing CsPbBr_3_/APTES/toluene, then precipitation of this solution with methyl acetate solvent obtained CsPbBr_3_@SiO_2_ nanoparticles. The shell thickness increased from 1.5 to 3.5 to 4.2 nm as hydrolysis time increased from 5 to 10 to 30 min. However, if hydrolysis exceeded 1 h, serious aggregation was observed due to excessive APTES hydrolysing to form bulk SiO_2_. Thanks to the shell formed on the surface of CsPbBr_3_, CsPbBr_3_@SiO_2_ still showed bright emission in water for 48 h. By contrast, CsPbBr_3_ without a SiO_2_ shell was highly unstable in a water environment. Its PL features completely disappeared in water after a duration of less than 1 h. The CsPbBr_3_@SiO_2_ nanoparticles exhibited great photoluminescence, high water stability, biocompatibility characteristics and low cytotoxicity. These CsPbBr_3_@SiO_2_ nanoparticles were used as the fluorescent probe for CT26 tumour cell imaging in this work. This research showed the potential of lead halide perovskites core–shell for tumour diagnosis, for the new generation of optical probes in various biomedical applications such as cancer cells.

Different from the synthesis methods mentioned above, Zang *et al*. [63] reported one-step room-temperature synthesis to coat CsPbBr_3_ QDs with APTES. This entire process only took a short time (20 s) at room temperature. This method was simple and did not require additional water or prolonged stirring. Therefore, the QDs will be prevented from decomposing by water before SiO_2_ formation. The solution containing PbBr_2_, CsBr, OA and OAm was quickly added to toluene containing APTES at room temperature. During the hydrolysis of APTES, an SiO_2_ shell was formed via the interaction between QDs surfaces and APTES. XRD patterns showed that the CsPbBr_3_@SiO_2_ QDs exhibited an orthorhombic structure, similar to that of CsPbBr_3_ QDs. This proved that the SiO_2_ shell has no influence on the crystallinity of CsPbBr_3_ QDs. In addition, the CsPbBr_3_@SiO_2_ QDs also exhibited excellent stability against oxygen, moisture, heat and polar solvent (ethanol). The stability of CsPbBr_3_ QDs and CsPbBr_3_@SiO_2_ QDs films was tested under heating temperature at 80°C. The PL intensity of the CsPbBr_3_@SiO_2_ QDs slowly decreased and still remained at 53% value of PL intensity after 60 min of heating at 80°C. By contrast, CsPbBr_3_ QDs rapidly declined after a duration of 60 min. Similar to the stability under heating, after 30 min with ethanol, CsPbBr_3_@SiO_2_ QDs still maintained 87% of PL intensity, whereas the bright emission of CsPbBr_3_ QDs disappeared. This confirmed that SiO_2_-coated CsPbBr_3_ improved the resistance to heat and high chemical stability due to the protection of the SiO_2_ shell on the surface of QDs. The CsPbBr_3_@SiO_2_ QD was used as a photonic material with red Ag–In–Zn–S QDs on InGaN blue chips to fabricate WLEDs. These WLEDs exhibited excellent luminescent performance with a power efficiency of 40.6 Lm W^−1^. This work provided SiO_2_-coated CsPbBr_3_ core–shell perovskite QDs as potential photonic materials with high PLQY, and good chemical, as well as stability, which are suitable for highly efficient (high colour-rendering thermal index) and stable WLEDs devices. This demonstrates their potential applications in display and solid-state lighting fields.

Furthermore, Wei *et al*. group [64] reported a modified ligand prepared from bis[3-(triethoxysilyl) propyl] amine with gultaric anhydride (BTPA-GA) and APTES to passivate the CsPbBr_3_@SiO_2_ QDs surface. After the QDs were dispersed in a polystyrene (PS) matrix and under heat treatment, Si-O-Si cross-linking of the surface ligands was formed to protect CsPbBr_3_. The PS matrix was used to interweave the CPB@SiO_2_ and prevent aggregation during the formation of CPB@SiO_2_. The CPB@SiO_2_/PS film was dissolved in toluene and washed three times to remove the PS matrix. A cross-linked Si-O-Si organic silica network formed from the hydrolysis process of ethoxyl silane ether groups and condensation reaction under heating. Meanwhile, the –NH_2_ group of APTES also controlled the size of QDs. In addition, the –COOH group of BTPA-GA suppressed the aggregation of QDs and affected the coordination between the –COOH group and Pb. The size of CsPbBr_3_ QDs without PS matrix was larger and exhibited a more irregular morphology compared with that of the CPB QDs. Introducing a SiO_2_ protective layer onto the surface of CPB@SiO_2_ enhances its stability when exposed to acid-base environments and polar solvents. The PL peak intensity of CPB@SiO_2_ did not change much in DMF over the duration of one month. In addition, CPB@SiO_2_ still survived in ammonium hydroxide or tetrabutylammonium hydroxide, or acid (acetic). Owing to excellent properties of stability in polar solvents, water and acid-alkaline conditions, this material was used as solution-processable luminescent inks. A five-letter ‘ECUST' pattern written using CsPbBr_3_@SiO_2_ solution in toluene as ink exhibited excellent uniform, strong PL emission and survived until 100 s. By contrast, the ‘ECUST' letter pattern was prepared from SiO_2_-uncoated CsPbBr_3_ solution as the ink faded. This work suggested a new method to produce core–shell perovskites with excellent stability in various conditions and became promising materials in the ink field. In addition to bio and optoelectronics applications, perovskite@SiO_2_ with excellent luminescence performance, stability and dispersion in ethanol was applied in sensing applications. Particularly, in Chen *et al*. group [65], CsPbBr_3_@SiO_2_ PNCs were used in the fluorescence sensing of Cl^−^ in sea sand samples. This group successfully developed CsPbBr_3_@SiO_2_ PNCs by an efficient synthesis method with the support of a nucleophilic substitution strategy using benzylic bromide. This process opens up new possibilities for the expansion of perovskite@SiO_2_ nanocrystals-based fluorescent sensors.

## 4. Conclusion

The lead halide perovskite materials-based applications have drawn the attention of scientists for stable inorganic halide perovskites and high performance of the device. The lead halide perovskites have excellent optical properties, which will be useful for optoelectrical and bio-related fields. However, their stability against external factors such as environment or polar solvents is very poor. This limitation has hindered the practical applications of these materials. Therefore, various materials containing SiO_2_ groups, such as TMOS, TEOS and APTES, were introduced to stabilize the perovskite. The encapsulation of SiO_2_ as the shell helps dramatically improve the endurance of perovskites under environmental and/or chemical conditions. This also helps increase their high PLQY with narrow sizes. In addition, the shell thickness can be well controlled so that these materials can be applied for various applications such as PeLED, WLED, bio-imaging and drug delivery. Enhancement of environmental and chemical stability of inorganic perovskites as well as controlling the thickness shell of inorganic halide perovskites core–shell play an important role in developing new types of halide perovskites for practical applications. This review will be useful in guiding researchers to find the suitable method for fabricating stable and highly efficient perovskite core–shell-based devices.

However, there are still some remaining limitations of SiO_2_-coated lead halide perovskite core–shells:

1. Conditions applied in the process of the growth of the shell by hot injection method often need an inert environment, high temperature, precise timing and sophisticated equipment. Even though this method can produce uniform perovskite core–shell, this requirement is a barrier to commercialization due to the less effectiveness during mass-scale production.
2. Heavy metals from the decomposition of the perovskites@SiO_2_ after long-term storage in the air will affect the environment. This will limit their applications (bio-related fields, etc.).
3. Perovskite core–shell shows great potential in many applications (optoelectronic, bio-imaging, etc.). But, the separation and extraction of excited charge carriers can be inhibited due to their own structure. This will hinder the material in some applications such as solar cells. Variety of new perovskites core–shell should be studied.

Thus, we suggest a synthetic process that involves lead halide perovskite core–shell with greener solvents, instead of common organic solvents during the formation of the SiO_2_ protective layer and device fabrication. Furthermore, we can also apply this type of silica-based core–shell structure to halide lead-free perovskites, which will not be harmful to human health and the environment. This will open new chances for the development of eco-friendly perovskite materials in bio-related, optoelectronic and other related fields.

## Data Availability

This article has no additional data.
